# The non-genomic loss of function of tumor suppressors: an essential role in the pathogenesis of chronic myeloid leukemia chronic phase

**DOI:** 10.1186/s12885-016-2346-6

**Published:** 2016-05-16

**Authors:** Sabrina Crivellaro, Giovanna Carrà, Cristina Panuzzo, Riccardo Taulli, Angelo Guerrasio, Giuseppe Saglio, Alessandro Morotti

**Affiliations:** Department of Clinical and Biological Sciences, University of Turin, San Luigi Hospital, Regione Gonzole 10, 10043 Orbassano, Italy; Department of Oncology, University of Turin, Orbassano, Italy

**Keywords:** Chronic myeloid leukemia, Tumor suppressor, Tyrosine kinase inhibitors, Non genomic loss of function

## Abstract

**Background:**

Chronic Myeloid Leukemia was always referred as a unique cancer due to the apparent independence from tumor suppressors’ deletions/mutations in the early stages of the disease. However, it is now well documented that even genetically wild-type tumor suppressors can be involved in tumorigenesis, when functionally inactivated. In particular, tumor suppressors’ functions can be impaired by subtle variations of protein levels, changes in cellular compartmentalization and post-transcriptional/post-translational modifications, such as phosphorylation, acetylation, ubiquitination and sumoylation. Notably, tumor suppressors inactivation offers challenging therapeutic opportunities. The reactivation of an inactive and genetically wild-type tumor suppressor could indeed promote selective apoptosis of cancer cells without affecting normal cells.

**Main body:**

Chronic Myeloid Leukemia (CML) could be considered as the paradigm for non-genomic loss of function of tumor suppressors due to the ability of BCR-ABL to directly promote functionally inactivation of several tumor suppressors.

**Short conclusion:**

In this review we will describe new insights on the role of FoxO, PP2A, p27, BLK, PTEN and other tumor suppressors in CML pathogenesis. Finally, we will describe strategies to promote tumor suppressors reactivation in CML.

## Background

Chronic Myeloid Leukemia (CML) was generally referred as an unique cancer, due to the apparent independence from tumor suppressors’ deletions/mutations in the early stages of the disease [1]. In agreement with this concept, infection of murine stem cells with BCR-ABL-expressing vectors was also associated with rapid development of CML without the need of additional genetic lesions [2]. Over the last few years, the involvement of tumor suppressors (TS) in cancer pathogenesis has been completely revised [3–5]. In particular, while in the original Knudson’s model TS are involved in tumorigenesis upon inactivation of both alleles (generally one through point mutation and one through deletion), it is now clear that even genetically wild-type TS can modulate tumorigenesis when down-regulated, aberrantly compartmentalized and/or affected by phosphorylation/acetylation/ubiquitination and others post transcriptional modifications.

In line with these observations, CML could represent the paradigm of how cancer can arise upon functional inactivation of tumor suppressors. In this review, we will describe how BCR-ABL directly promotes TS inactivation with important therapeutic implications. Finally, we will also describe those TS that are inactive in CML but without a clear direct regulation by BCR-ABL.

## Tumor suppressors directly inactivated by BCR-ABL

### FoxO

**Fig. 1:**
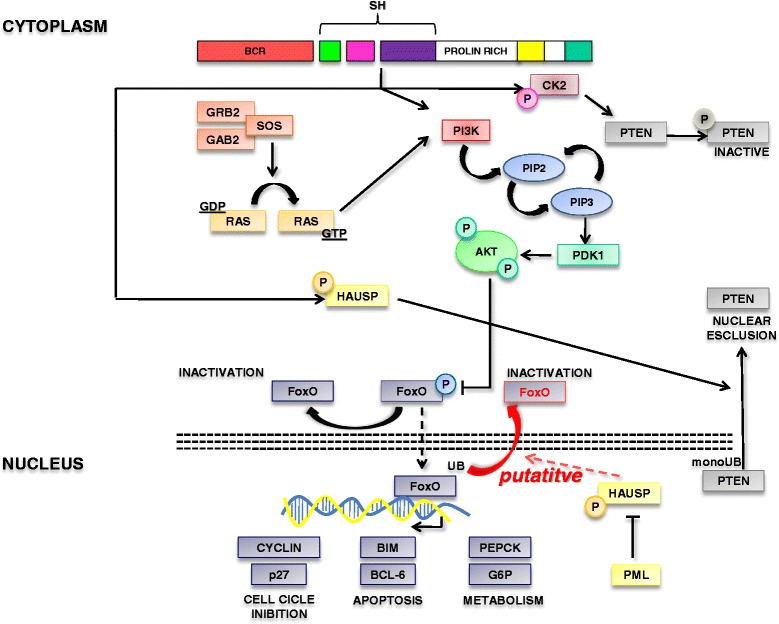
Tumor suppressors network associated with the BCR-ABL/PI3K/AKT pathway. Schematic representation of the BCR-ABL/PI3K/AKT pathway and the role of PTEN and FOXO tumor suppressors. This carton highlights how BCR-ABL inactivates PTEN through CKII-mediated phosphorylation and HAUSP-mediated changes of cellular compartmentalization. Furthermore, BCR-ABL promotes FOXO inactivation through the regulation of its cellular localization. We also speculate on the putative regulation of FOXO localization through HAUSP in CML, although BCR-ABL/HAUSP/FOXO connection has to be demonstrated

The Forkhead box subgroup O (FoxO) family of transcription factors (TFs) is a subclass of Forkhead transcription factors characterized by a winged helix DNA binding domain known as a Forkhead box [6, 7]. This family comprises four members (FoxO1, FoxO3, FoxO4 and FoxO6). In the presence of several Growth Factors (GFs) or activated tyrosine kinases, the PI3K-AKT signal transduction pathway promotes FoxO phosphorylation, favoring nuclear exclusion and suppression of transcriptional activity. Conversely, in the absence of GFs, un-phosphorylated FoxOs translocate into the nucleus where they modulate the expression of several genes. Furthermore, FoxOs are regulated by several protein modifications, such as acetylation, ubiquitination and arginine/lysine methylation. FoxOs have been described as essential components of BCR-ABL signal transduction [8–10]. In particular, BCR-ABL is a strong activator of the PI3K-AKT pathway and therefore promotes the inactivation of FoxO3a, FoxO1 and FoxO4 though phosphorylation and shuttling into the cytoplasm. On the contrary, Tyrosine Kinase Inhibitor (TKI) treatment promotes the reactivation of FoxOs which in turn are able to mediate cell cycle arrest. Reactivation of FoxOs is associated with the down-regulation of CCND1/Cyclin D1 protein expression and affects the expression of stem cell genes such as ATM, p57/CDKN1C, and BCL6 [10]. Similarly, another report highlighted BCL6 as an essential FoxO downstream mediator of cell renewal [11]. As a consequence, FoxOs reactivation impacts on the maintenance of the leukemia stem cells without affecting the normal hemopoietic stem cell compartment. Other authors have also shown that TGF-beta is involved in the regulation of FoxOs with consequent regulation of the LSC compartment [9, 12]. Notably, the BCR ABL/PI3K/AKT/FoxO pathway is less dependent on BCR-ABL activity in the stem cell compartment [10]. This finding could explain the reason why stem cells remain quiescence even in the presence of BCR-ABL and are resistant to TKI treatment. The mechanism of FoxOs nuclear retention in stem cells is still not explained in detail, although it was associated with AKT-mediated phosphorylation. Since FoxOs localization is also regulated by mono-ubiquitination [13] and that BCR-ABL activates the FoxOs-deubiquitinase HAUSP [14], it could be speculated that FoxOs nuclear localization could be affected by BCR-ABL/PML/HAUSP network in a similar manner as for PTEN [14]. However, experimental studies are mandatory to demonstrate this network with important therapeutic implications, due to the availability of HAUSP inhibitors (Fig. 1).

### PP2A

**Fig. 2:**
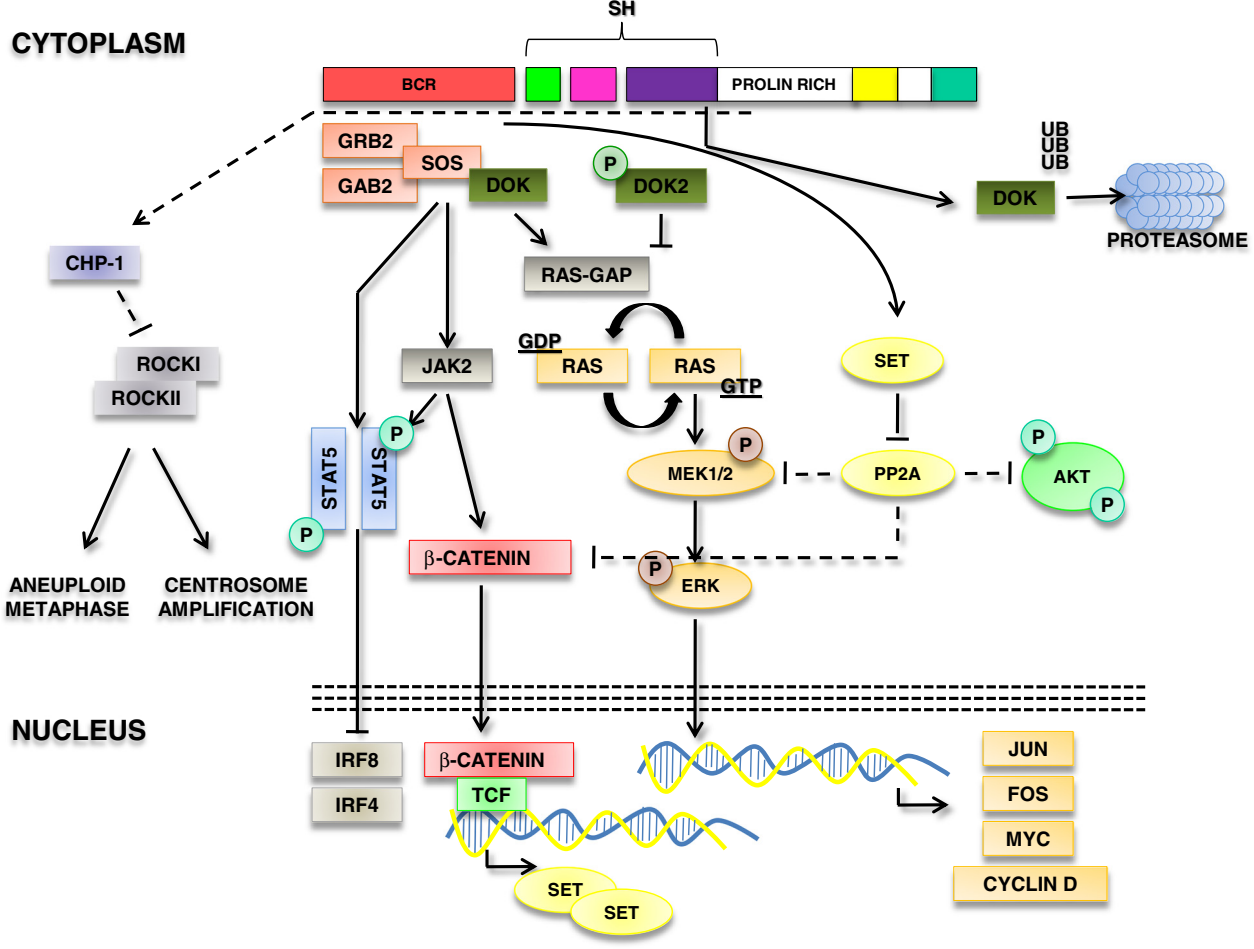
Tumor suppressors network associated with the BCR-ABL/MAPK pathway. Representation of the tumor suppressors involved in the RAS/MEK/ERK pathway and the JAK2/β-catenin pathway. DOK family proteins are involved in the negative regulation of RAS activation. Representation of the IRF pathway. Finally, morgana/chp-1 pathway is indicated

In the last years, it was demonstrated that BCR-ABL, irrespectively to its tyrosine kinase activity, is able to promote the recruitment and the activation of the Janus kinase 2 (JAK2) [15]. JAK2 is in turn able to enhance β-catenin activity which is responsible of SET-mediated inactivation of protein phosphatase 2A (PP2A). PP2A is a ubiquitous serine/threonine phosphatase that targets Raf, MEK, AKT and other essential mediators of oncogenic signals [16]. Besides having linked β-catenin signaling to the inactivation of a tumor suppressor, the relevance of these observations relies on the fact that PP2A activity can be restored by PP2A activating drugs [17]. In particular, the orally available FTY720 promotes the activation of PP2A favoring CML cells and CML stem cells apoptosis [18, 19]. Most importantly, this drug was shown to induce apoptosis in the tyrosine kinase resistant stem cell pool [19] (Fig. 2).

### p27

**Fig. 3:**
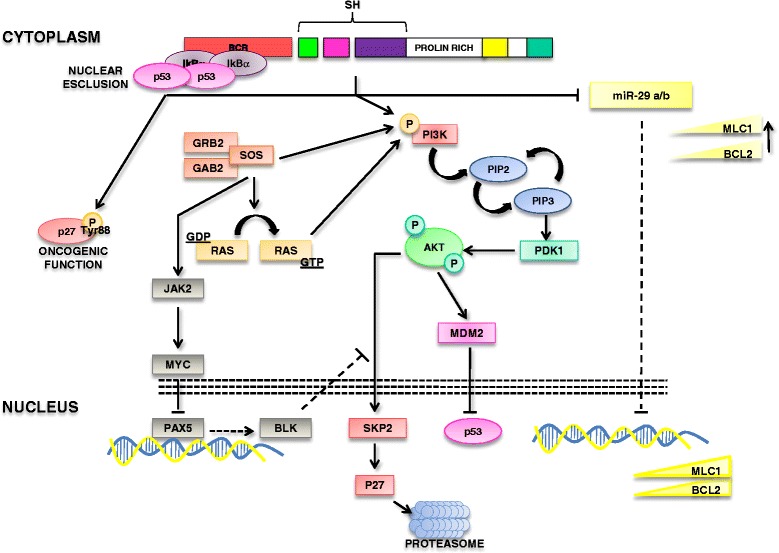
BCR-ABL/p53 connection, p27 network and miRNAs in CML. BCR-ABL promotes either sequestration of p53 in the cytoplasm through the interaction with IkB-alpha and the p53 degradation through MDM2. Furthermore, the interaction BCR-ABL with p27 is reported

p27 is an inhibitor of cyclin-dependent kinases (Cdk2) involved in the control of cell-cycle [20]. As most of the regulator of cell-cycle, p27 is tightly regulated at different levels. P27 has been referred as a tumor suppressor, although a paradoxical dual role (oncogenic/tumor suppressor role) has been postulated. Notably, changes in p27 cellular compartmentalization appears to play an essential role in tumorigenesis: nuclear exclusion was indeed associated with adverse prognosis in several cancers [21]. BCR-ABL was shown to regulate p27 at different levels. In particular, BCR-ABL affects p27 expression and promotes degradation of nuclear p27 [22–25]. Moreover, BCR-ABL promotes FoxO3a inhibition through PI3K-AKT with consequent impairment of p27 transcription. Furthermore, PI3K regulates the activity of SKP2 which mediates p27 degradation. BCR-ABL is also able to promote p27 phosphorylation on tyrosine 88 which is involved in the control of cyclinE/Cdk2 activity. More recently, BCR-ABL was shown to promote oncogenic gain of functions of cytoplasmic p27 [26]. The overall role of p27 in CML pathogenesis is that nuclear p27 acts as a tumor suppressor promoting cell cycle regulation; on the contrary, cytoplasmic p27 is acting as an oncogene. The relevance of p27 network relies on the fact that forcing p27 into the nucleus can dictate cancer selective growth arrest and apoptosis [26] (Fig. 3).

### PTEN

The tumor suppressor PTEN is involved in either the regulation of the PI3K-AKT pathway and phosphatase independent functions [27]. Several recent reports have demonstrated that PTEN plays an essential role in the pathogenesis of CML [28], as reviewed elsewhere [29]. In particular, PTEN was reported to be i) under-expressed through a Ras-MEK pathway [30, 31], ii) inactivated through tail phosphorylation [32] and iii) delocalized into the cytoplasm [14] (Fig. 1).

### DOK genes

DOK1 and DOK2 are rasGAP-associated docking proteins, that are preferentially expressed in the hematopoietic cells, and behave as tumor suppressors in both myeloproliferative disorders and lung cancer [33]. DOK proteins contain a NH2-terminal Pleckstrin homology domain (PH), a Phosphotyrosine-binding domain (PTB) and a COOH-terminal SH2 target motif. DOK proteins bind to p120 rasGAP and therefore counteract the activation of the RAS-RAF-MEK pathway. DOK1, also known as p62dok, and DOK2 were originally cloned as a BCR-ABL substrate in CML [34–37]. Expression of both Dok1 and Dok2 opposes BCR-ABL mediated leukemogenesis [38, 39]. Although DOK1 and DOK2 have not been extensively studied in human CML samples, it was demonstrated that DOK phosphorylation by BCR-ABL is associated with the inactivation of its activity as a Ras-GAP [40]. Furthermore, BCR-ABL was also shown to promote DOK1 ubiquitination and degradation [41]. All together, these data indicate that DOK proteins act as tumor suppressors through the inhibition of the RAS-MEK-ERK pathway, but in CML their function is directly inhibited by BCR-ABL (Fig. 2).

### p53

TP53 is a tumor suppressor exerting a pivotal role for the maintenance of genomic integrity in response to several cellular stresses [42]. According to the damage, p53 induces the transcription of several genes that block cell cycle, or that promote apoptosis, like p21/WAF1 and Bax [43]. The p53 protein is generally expressed at low levels in normal cells and has a short half-life [44]. P53 function is counteracted by MDM2 oncoprotein, that by binding the p53 transactivation domain, inhibits its transcriptional activity, and promotes p53 nuclear export. Moreover, MDM2 acts like a E3 ubiquitin ligase, mediating p53 degradation in a proteasome-dependent manner. Furthermore MDM2 gene is a direct transcriptional target of p53, thereby p53 and MDM2 form a negative feedback loop where p53 regulates the expression of MDM2, that in turn blocks p53 functions and promotes its degradation. The tumor suppressor TP53 plays an essential role in the pathogenesis of several cancers. Within myeloid malignancies, TP53 was also implicated in the progression of CML into the blast phase [45]. In particular, almost 20 % of CML blast phases express TP53 mutations, but no mutations/deletions were reported in the chronic phase of CML. Although un-mutated and not deleted, p53 is functionally inactivated in the chronic phase of CML patients [46]. A mechanism described by Calabretta’s group shows that BCR-ABL upregulates the expression of MDM2 by increasing its translation that is dependent on high levels of the La antigen, an RNA binding protein. The BCR-ABL/MDM2 regulation could indeed affect p53 function. P53 activity could also be regulated by the phospho-status of its negative regulator MDM2. AKT-mediated phosphorylation of MDM2 promotes its nuclear localization that favors the inhibition of p53 [47]. Recently, we have shown that BCR-ABL is able to stabilize an IkB-alpha/p53 complex which is responsible for the sequestration of p53 into the cytoplasm of CML cells [48]. In particular, the NF-kB inhibitor IkB-alpha is able to interact with either NF-kB p65 subunit or the p53 protein. This complex prevents p53 to interact with DNA response elements and to promote apoptosis. Notably, BCR-ABL is able to interact and stabilize IkB-alpha in the cytoplasm therefore promoting p53 sequestration into the cytosol. As a consequence, IkB-alpha prevents p53 mediated apoptosis (Fig. 3).

### IRF-8 and IRF-4

The interferon regulatory factor-8 (IRF-8) is an essential myeloid transcription factor involved in the regulation of the myeloid lineage commitment [49]. Notably, IRF-8 deletion in the mouse is associated with the development of CML like MPD [50]. Interestingly, IRF-8 is under-expressed in CML [51]. BCR-ABL activates STAT5 which in turn represses IRF-8. The BCR-ABL/STAT5/IRF-8 network is another example of the BCR-ABL ability to promote tumor suppressors inactivation. Similarly, BCR-ABL regulates the function of another interferon regulatory factor (IRF-4), suggesting that these transcription factors are downstream effectors of the chimeric translocation [52] (Fig. 2).

### BCR-ABL/oncogenic miRNA mediated tumor suppressors down-regulation

The involvement of miRNAs in CML pathogenesis is highly complex and include both oncogenic miRNA and tumor suppressive miRNAs [53]. In line with the aim of this review, it should be noted that BCR-ABL is able to positively regulate several oncogenic miRNAs which in turn affect the expression of tumor suppressors [54], with consequent inactivation.

In line with these considerations, for instance, BCR-ABL is able to regulate the expression of oncogenic miR-130a and miR-130b which in turn affect the expression of the tumor suppressor CCN3 [55].

## Tumor suppressors, involved in CML pathogenesis, not directly regulated by BCR-ABL

In this section, we will report on tumor suppressors that have been described as inactive in CML, although in a genetically wild-type status. In particular, we focus on those tumor suppressors that are not directly regulated by BCR-ABL but that cooperate with BCR-ABL in the development of CML.

### Morgana/chp-1

Morgana/chp-1 is a chaperon protein involved in the regulation of centrosome duplication and genomic stability [56]. Morgana forms a complex with ROCKI and ROCKII promoting the inhibition of their kinase activity and therefore suppressing centrosome over-duplication. We have recently demonstrated that morgana haploinsufficiency is associated with the development of a transplantable myeloproliferative disorder [57]. Furthermore, we have observed that a portion of CML exhibits morgana under-expression, which is associated with increased centrosome amplification and aneuploid metaphases. Notably, patients expressing low levels of morgana are associated with a worse response to TKIs. Due to the ability of morgana to regulate ROCK activity, the sensitivity to TKI of cell obtained from morgana underexpressing patients can be rescued by treating cells with ROCK inhibitors. These data suggest that morgana/chp-1 can cooperate with BCR-ABL in the pathogenesis of CML and in the development of TKI resistant CML. However, the mechanisms of morgana downregulation in CML still have to be clarified (Fig. 2).

### BLK

By using microarray analyses of leukemic stem cells, it was showed that the *Blk* gene is markedly down-regulated in the CML stem cell pool. BLK expression was dependent on BCR-ABL protein but independent of its kinase activity [58]. Notably, Blk was shown to be involved in the regulation of Leukemic stem cells maintenance. Blk is a member of the Src tyrosine kinase. Although Src proteins behave as oncogenes, Blk was shown to act as a tumor suppressor through the regulation of CML cells proliferation, in a pathway involving c-myc and p27 (Fig. 3).

### Tumor suppressive miRNAs

Various miRNAs with known tumor suppressive roles have been found de-regulated in CML. In particular, miR-29a and miR-29b were shown to be down-modulated in CML and anti-correlated with the expression levels of target genes, Bcl-2 and Mcl-1 [59]. Interestingly, miR-424 and miR-320a that directly target the 3′UTR of the ABL gene are under-expressed in CML and miR-320a is also downregulated in CML cancer stem cells [60, 61]. Up-regulation of these miRNAs inhibits cell proliferation, induces apoptosis and, in the specific case of miR-424, also increases the sensibility to the Imatinib treatment. Others miRNAs have also been involved in the pathogenesis of CML [61, 62]. However, it should be noted that further analyses should be performed to address the mechanisms of miRNA deregulation in CML and the real *in vivo* contribution in CML pathogenesis (Fig. 3).

### PML

The tumor suppressor PML plays an essential role in the regulation of CML stem cell [63], and various reviews have been published on this topic [64, 65]. Furthermore, PML plays an essential role in the regulation of the tumor suppressive function of PTEN, through HAUSP [66]. The tumor suppressive functions of PML in CML are associated with the differential PML expression during the leukemic differentiation. While PML retains high levels of expression in the stem cell compartment, where it mediates stem cell quiescence, PML levels progressively drop during differentiation into progenitor and terminally differentiated cells. As a consequence, loss of PML is associated with both increased proliferation [63] and PTEN nuclear pool exclusion [14]. While the mechanism of PML tumor suppressive functions in CML are highly complex, it should be noted that PML is a targetable tumor suppressor due to the ability of arsenic trioxide to promote its degradation. Even if apparently contradictory in the context of cancer therapy, the degradation of PML promotes cell cycle induction of CML stem cells with consequent their exhaustion. PML targeting strategies offer the chance to achieve the eradication of CML [63].

## Strategies to promote tumor suppressor reactivation

The inability to overcome genetic inactivation of tumor suppressors with anticancer therapies is currently challenging. Conversely, targeting mechanisms implicated in non genomic tumor suppressor loss of function could become a new potential strategy to enhance or support target therapy responses. In particular, inhibitors of CKII are able to promote PTEN tumor suppressive functions [32]. Accordingly, HAUSP inhibitors, as well as arsenic trioxide [63] could restore PTEN nuclear localization with pro-apoptotic and antiproliferative effects. Similarly, reactivation of PP2A was show to antagonize oncogenic BCR-ABL properties *in vitro* [17]. Direct pharmacological activation of PP2A by Forskolin, or indirect targeting of inhibitor components of PPA2 pathway (such as SET inhibitors) reduced proliferation and clonogenic potential, and induced apoptosis in myeloid malignances [19]. The loss of a tumor suppressor gene can also cause the activation of a side pathway. This is what happens in CML patients expressing low levels of Morgana. The increase of ROCK activity consequent to Morgana modulation confers imatinib resistance. Treatment with ROCK inhibitor was shown to rescue the apoptotic response to imatinib [57].

## Discussion

The oncogenic BCR-ABL signal is part of a complex network of interactions that mediate proliferation and survival. Parallel to these signaling transduction pathways, BCR-ABL is also able to mediate the inactivation of several tumor suppressors, through either i) regulation of gene expression, or ii) changes in cellular compartmentalization or iii) directly or indirectly, favoring protein modifications, such as phosphorylation/ubiquitination/acetylation. The relevance of these networks relies on the fact that targeting mechanisms that promote tumor suppressors inactivation can restore their function with consequent strong and selective cancer apoptosis. In our opinion, the development of strategies to reactivate tumor suppressors is a really challenging therapeutic option and CML could represent an essential model to verify the efficacy of this novel targeted molecular therapy. In particular, those cases characterized by resistance to TKI could benefit with combined therapy to achieve synthetic lethality [67].

## Conclusion

CML chronic phase is not associated with known TS genetic loss of function, suggesting that BCR-ABL is sufficient for the development of this disease. However, as we have reviewed here, BCR-ABL has the ability to functionally inactivate several tumor suppressors allowing to promote tumorigenesis through an highly complex signal transduction network. The functional inactivation of TS is a great opportunity to design combinatorial therapies to achieve synthetic lethality together with BCR-ABL tyrosine kinase inhibitors.

## Abbreviations

CML: chronic myeloid leukemia
TS: tumor suppressors
FoxO: Forkhead box subgroup O
GF: growth factors
TKI: tyrosine kinase inhibitors
JAK2: Janus kinase 2
PTB: phosphotyrosine-binding domain
IRF-8: interferon regulatory factor-8
